# Using life‐history trait variation to inform ecological risk assessments for threatened and endangered plant species

**DOI:** 10.1002/ieam.4615

**Published:** 2022-05-24

**Authors:** Pamela Rueda‐Cediel, Nika Galic, Richard Brain, Jesús N. Pinto‐Ledezma, Andreu Rico, Valery Forbes

**Affiliations:** ^1^ Department of Ecology, Evolution, and Behavior University of Minnesota St. Paul Minnesota USA; ^2^ Syngenta Crop Protection LLC Greensboro North Carolina USA; ^3^ IMDEA Water Institute, Science and Technology Campus of the University of Alcalá Alcalá de Henares Madrid Spain; ^4^ Cavanilles Institute of Biodiversity and Evolutionary Biology University of Valencia Paterna Valencia Spain

**Keywords:** Elasticity, Endangered Species Act, Matrix population models, Pesticides, Phylogeny

## Abstract

Developing population models for assessing risks to terrestrial plant species listed as threatened or endangered under the Endangered Species Act (ESA) is challenging given a paucity of data on their life histories. The purpose of this study was to develop a novel approach for identifying relatively data‐rich nonlisted species that could serve as representatives for species listed under the ESA in the development of population models to inform risk assessments. We used the USDA PLANTS Database, which provides data on plants present in the US territories, to create a list of herbaceous plants. A total of 8742 species was obtained, of which 344 were listed under the ESA. Using the most up‐to‐date phylogeny for vascular plants in combination with a database of matrix population models for plants (COMPADRE) and cluster analyses, we investigated how listed species were distributed across the plant phylogeny, grouped listed and nonlisted species according to their life history, and identified the traits distinguishing the clusters. We performed elasticity analyses to determine the relative sensitivity of population growth rate to perturbations of species' survival, growth, and reproduction and compared these across clusters and between listed and nonlisted species. We found that listed species were distributed widely across the plant phylogeny as well as clusters, suggesting that listed species do not share a common evolution or life‐history characteristics that would make them uniquely vulnerable. Lifespan and age at maturity were more important for distinguishing clusters than were reproductive traits. For clusters that were intermediate in their lifespan, listed and nonlisted species responded similarly to perturbations of their life histories. However, for clusters at either extreme of lifespan, the response to survival perturbations varied depending on conservation status. These results can be used to guide the choice of representative species for population model development in the context of ecological risk assessment. *Integr Environ Assess Manag* 2023;19:213–223. © 2022 The Authors. *Integrated Environmental Assessment and Management* published by Wiley Periodicals LLC on behalf of Society of Environmental Toxicology & Chemistry (SETAC).

## KEY POINTS

- Population models of US herbaceous terrestrial plants of no conservation concern may be representative of plants listed in the Endangered Species Act (ESA) and can help to inform risk assessments.
- Basing the selection of representative species in lieu of data on systematic life‐history analyses helps to ensure that population models representing listed species are both representative and protective.
- US herbaceous terrestrial plants (listed and nonlisted in the US ESA) exhibit clear differences in their age at first reproduction and longevity but not in their reproductive output.
- US herbaceous terrestrial plants that are long‐lived can be used as a sensitive representative for plants listed in the ESA when trying to evaluate the potential effects of herbicides that target plant survival.

## INTRODUCTION

Plant biodiversity is characterized by a great deal of life‐history variation. It is estimated that 15 447 vascular plant species occur in continental Canada and the United States, and 10 636 species are considered restricted to this geographical realm (Ulloa et al., 2017). The United States Department of Agriculture (USDA) PLANTS Database, which includes standardized information about the vascular plants, mosses, liverworts, hornworts, and lichens of the US and its territories, estimates that US flora, including Hawaii, Puerto Rico, and the Virgin Islands, contains 8742 species of herbaceous plants (USDA, NRCS, 2019). This plant richness represents several growth forms and life histories across the country. Out of that pool of species, 816 plant species are classified as threatened or endangered according to the US Endangered Species Act (ESA; https://ecos.fws.gov/ecp0/reports/ad-hoc-species-report?kingdom=P%26status=E%26status=T%26status=EmE%26status=EmT%26status=EXPE%26status=EXPN%26status=SAE%26status=SAT%26fcrithab=on%26fstatus=on%26fspecrule=on%26finvpop=on%26fgroup=on%26ffamily=on%26header=Listed%2BPlants, last accessed June 5, 2019) from here on referred to as “listed.” A vast majority of terrestrial listed species are herbaceous and exhibit potential spatial overlap with some agricultural crops or grow near agricultural lands. To comply with the ESA, pesticide registration in the United States under the Federal Insecticide, Fungicide, and Rodenticide Act (FIFRA; NRC, 2013) requires an evaluation of the potential risks posed to these listed species and their critical habitats. To facilitate this process, population models have been recommended as novel tools that integrate relevant pesticide exposure and life history data into a quantitative risk evaluation process (NRC, 2013).

Currently, there is only a handful of listed plant species population models, and these have mostly been applied to assess the impacts of habitat loss (Floy & Ranker, 1998; Rae & Ebert, 2002), and other natural stressors (e.g., fire and flood hazard; Kaye et al., 2001; Regan et al., 2010; Smith et al., 2005). Notably, a smaller set of models has been developed and applied to assess potential risks to plants from pesticide exposures (e.g., Crone et al., 2009; Schmolke, Brain, et al., 2017; Schmolke, Kapo, et al., 2017; Schmolke, Brain, et al., 2018; Schmolke, Roy, et al., 2018; Reeg et al., 2017; Reeg, Heine, Mihan, McGee, et al., 2018; Reeg, Heine, Mihan, Preuss, et al., 2018), yet the development of population models for listed plant species is still lacking (Forbes et al., 2016). The major limitation for model development is the scarcity of essential information regarding the biology and natural history of listed species, thus limiting the use of population models for listed species risk assessments as recommended by the National Research Council (NRC, 2013). Furthermore, legal restrictions prevent data collection and experimentation on listed species (Forbes et al., 2015). However, both threatened and nonthreatened plants are impacted by the same factors (agriculture being one of the most important; IUCN Red List 2016). A recent study has shown that population growth rates of listed and nonlisted plants exhibit similar responses to perturbations of their life‐history traits (Rueda‐Cediel et al., 2019). This raises the possibility of identifying representative species for the development of population models based on data‐rich nonlisted species to infer and assess risk to data‐poor listed species, like the “Robin Hood” approach for fish stock assessment (Punt et al., 2011). Given that it would not be feasible to develop unique population models for every single species, being able to identify a handful of representative species having sufficient data for modeling and that could represent broader groups of species is a practical way to proceed.

A better understanding of the similarities and differences in responses of listed and nonlisted species to perturbations (i.e., from pesticides and other human impacts) would facilitate the identification of representative species for modeling and inform both assessments of risks and choice of effective management options. In this study, we used ordination and phylogenetic analyses across terrestrial herbaceous plants of different conservation statuses to characterize their demography and vulnerability to stressors that can potentially impact their survival, growth, or reproduction. The goal was to use these analyses to inform the selection of data‐rich, nonlisted species that could potentially be used as representative species for population modeling in the context of pesticide risk assessment.

## METHODS

### General approach

We first distinguished US‐listed and nonlisted herbaceous plants. We then determined for which of these species a matrix population model was available, and we used it to extract life‐history traits for each species. Using the extracted life‐history traits we developed a hierarchical cluster to characterize similarities and differences within and across clusters and identified possible representative species for pesticide risk assessment. Since most of the published population models for plants use a matrix model structure (Crone et al., 2011)—and since there is a comprehensive database of matrix population models for plants (COMPADRE; Salguero‐Gómez et al., 2014)—we limited our analysis to matrix population models. Currently, in COMPADRE there are matrices for 36 US‐listed herbaceous species and 58 nonlisted herbaceous species, which have been modeled for conservation or theoretical purposes (Salguero‐Gómez et al., 2014). We performed elasticity analyses to estimate the effect of changes in various matrix elements (e.g., related to survival, growth, or fertility) on the asymptotic population growth rate (Caswell, 2001). In addition, we used the most up‐to‐date phylogeny for vascular plants (Smith & Brown, 2018) in combination with the COMPADRE database to determine how listed species, for which matrix population models are available, are distributed in the vascular plant phylogeny.

### Matrix population models for US herbaceous plants distributed phylogenetically

Data for herbaceous plants present in the United States, including Alaska, Puerto Rico, Hawaii, and the Virgin Islands, were downloaded from the USDA PLANTS Database (https://plants.sc.egov.usda.gov/java/, last accessed September 11, 2019). Subspecies, varieties, hybrids, and incomplete species were excluded from the data selection. We limited our sample to herbaceous species because this type of growth form tends to occur in proximity to agricultural crops. Only species that reported growth form, particularly forbs and herbs as the most frequent option, were selected. Species names were revised using the R package Taxonstand to retrieve the accepted scientific names according to The Plant List version 1.1 (http://www.theplantlist.org/). A total of 8742 species was obtained, out of which 344 species were listed under the ESA. Of the total pool of species, 86 were present in the COMPADRE database v.6.20.6.0, and 31 species were both listed and present in the COMPADRE database. We used the most up‐to‐date plant phylogeny (hereafter SB‐tree; Smith & Brown, 2018) to map the availability of matrix population models for herbaceous species in the United States. To do so, the SB‐tree was trimmed to include only the species sampled from the USDA PLANTS Database. Following Pinto‐Ledezma et al. (2020), missing species in the SB‐tree were added using taxonomic constraints in other words, missing species were added as terminal branches at the midpoint of their sister lineages.

### Extraction of life‐history traits from transition matrices

Projection matrices for herbaceous plants were collected from the COMPADRE database version 6.20.6.0. We used data entries that fulfilled the following conditions: (1) unmanipulated matrices or matrices that refer to natural conditions; (2) matrices that were decomposed in their survival and sexual reproduction components according to Caswell (2001) A=T+F, where A is the projection matrix that can be decomposed in the transition matrix, T, which includes survival, and the reproduction matrix, F, which includes sexual reproduction; (3) study duration of at least one year; (4) matrices with an annual periodicity; (5) studies that classified individuals based on their developmental stage or size; (6) matrices made from either the arithmetic mean of the element for several periods available, or pooled individual‐level data across populations and/or periods or from data for single study‐species‐treatment‐period combination; (7) matrix dimensions of at least 2 × 2 stages; (8) survival issue <1.05; this refers to the tolerable level of error for survival estimates (e.g., they should not add up to >1.05); and (9) irreducible matrices (i.e., matrices in which all stages are connected and contribute to any other stage). These conditions were set following the definitions of the COMPADRE User Guide (COMPADRE Plant Matrix Database, 2019). A total of 77 species, including 22 listed species, were obtained once all conditions were applied.

The projection matrices for each species were used to calculate the population growth rate (*λ*) and six demographic traits (longevity, $L_{\max}$, survivorship curve type, H, age at first reproduction, $L_{\alpha }$, mean life expectancy, $L_{\text{mean}}$, degree of iteroparity, S, and net reproductive rate, $R_{o}$). These traits were calculated using the Markov Chain Decomposition method where the time spent in each stage (N matrix) can be estimated from the T matrix, which also allows calculation of the life‐table elements, survivorship ($l_{x})$ and fertility ($m_{x})$ (Caswell, 2001). For this, we used the R package Rage (Jones & Salguero‐Gómez, 2020) that applies Caswell's methods to calculate age‐specific traits from stage‐specific models. Specifically, with the survivorship schedule, $L_{\max}$, the age at which survivorship drops to a critical point (0.01 default value) and $L_{\text{mean}}$ were calculated. Survivorship is the cumulative probability of surviving to a given age. When plotted on a log scale, three types of curves are observed; I (concave, with H>1), II (a straight line, with H=1), and III (convex, with H<1). This was calculated with the following equation:

(1) $$H=\frac{-\log(l_{x})l_{x}}{\sum l_{x}}$$

The net reproductive rate was calculated as the dominant eigenvalue R, which is the product of the matrices F and N. The degree of iteroparity (S), which describes the frequency of sexual reproductive events, was calculated with the following equation. According to Salguero‐Gómez et al. (2016), values of S≈0 correspond to highly semelparous species, and high values (S≫0) imply a high degree of iteroparity.

(2) $${S=-e}^{\log\lambda }l_{x}m_{x}\log(e^{-\log\lambda }l_{x}m_{x})$$

Once the six traits were calculated and compiled, the data were normalized by applying the following transformation:

(3) $$y_{\text{normalized}}=\frac{y_{i}-\min(y_{i})}{\left(y_{i}\right)-\min(y_{i})}$$

where i represents a given trait, y represents the value of the trait and min refers to the minimum value of the trait.

### Cluster analysis for US herbaceous plants

A hierarchical cluster analysis was performed with the trait data for 77 plant species from COMPADRE using the Bray–Curtis association distance coefficient matrix and the average linkage method (i.e., unweighted pair group method with arithmetic mean [UPGMA]). The choice of the cutting level in the dendrogram was determined by comparing cluster estimates generated from *K*‐means, which minimizes the within‐cluster variance and maximizes the between‐cluster variance (Everitt & Hothorn, 2011). The cluster analysis and the estimation of the cutting level were performed using the R packages vegan and cluster, respectively. Once the desired number of clusters was determined, a cutting point of 0.3 was established in the hierarchical cluster. A permutational multivariate analysis of variance (PERMANOVA) was conducted across the clusters obtained from the hierarchical clustering to quantify statistical differences between each cluster. To visualize which traits dominated in each cluster, a principal components analysis (PCA) was performed including the trait data for the evaluated species.

### Phylogenetic signal in demographic traits

We evaluated the phylogenetic signal in the six demographic traits using Pagel's *λ* (Pagel, 1999) under a Bayesian framework. Under Pagel's *λ*, traits evolve following a Brownian Motion (BM) model, that is, species trait values change randomly over evolutionary time. Values of Pagel's *λ* range from 0 (indicating phylogenetic independence or no phylogenetic signal) to 1, suggesting that traits evolved according to a BM model (phylogenetic dependence or phylogenetic signal). To estimate Pagel's *λ*, we ran Markov Chain Monte Carlo (MCMC) chains for 1 million generations and discarded 20% of the samples as burn‐ins in the R package motmot (Thomas & Freckleton, 2012). Convergence in the parameter estimate was assessed using Effective Sample Size (ESS). An ESS higher than 200 indicates that chains converged.

### Elasticity analysis across clusters

Elasticities were estimated as a proportional response of the population growth rate to a proportional change in each matrix element (Caswell,  2001). This analysis provides indications of the effect of a small change in each matrix element on the population growth rate. Elasticities were calculated using the R package Popbio (Stubben & Milligan, 2007) and Equation (4). Where $e_{\mathrm{ij}}$ denotes each elasticity element corresponding to the transition matrix element $a_{\mathrm{ij}}$ where the subindices ij refer to the matrix row and column, λ is the population growth rate, and ∂ indicates a partial derivative.

(4) $$e_{\mathrm{ij}}=\frac{a_{\mathrm{ij}}}{\lambda }\frac{\partial \lambda }{\partial a_{\mathrm{ij}}}$$

We calculated elasticities for each species that was included in the cluster analysis. Then we added up matrix elasticity elements for the processes of survival, growth, and reproduction, and compared them using density plots, which smooth the distribution of elasticity values across clusters. Elasticity comparisons were limited to the species belonging to Clusters 1–3, as the remaining clusters had too few species for a robust comparison.

## RESULTS

### Existing population models for US herbaceous plants distributed phylogenetically

**Figure 1:**
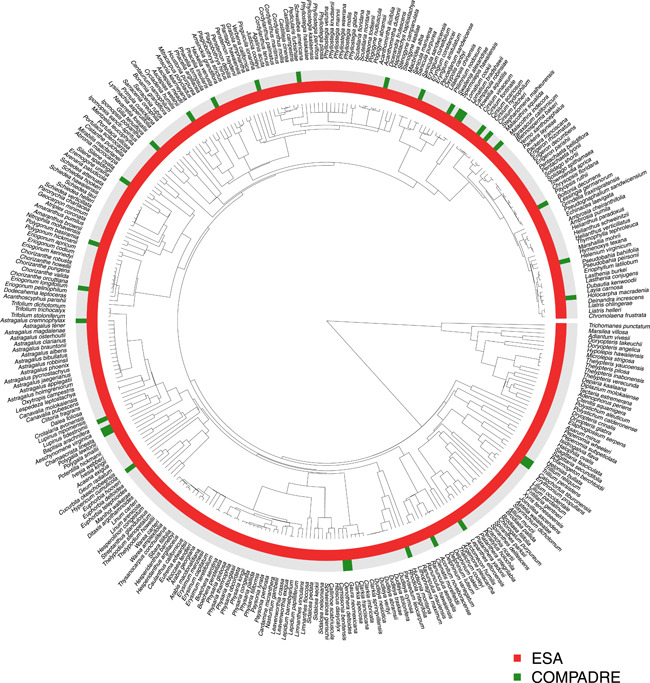
Phylogeny of listed species. Smith and Brown (2018) phylogeny was trimmed to include listed species and species present in COMPADRE. Red indicates the presence in the list of species listed under the Endangered Species Act (ESA). Green indicates the presence in the COMPADRE database

Overall, the phylogeny of US herbaceous species included 160 families; of those, 62 families contained listed species, 30 families contained species from COMPADRE, and 17 families contained species that were both present in COMPADRE and listed (Figures 1, S1). Listed species were widely and evenly distributed across the phylogeny. Listed species were more numerous across the following families: Compositae (3.26%), Brassicaceae (8.59%), Thelypteridaceae (6.56%), Malvaceae (8.96%), Dryopteridaceae (7.32%), Crassulaceae (12.33%), Asparagaceae (4.17%), and Apocynaceae (4.71%), compared to the rest of the clades (Figure S1). The numbers within brackets next to each family correspond to the percentage of listed species within that family. In contrast, species included in COMPADRE were less evenly distributed in the phylogeny; for instance, several listed species belonging to the families Brassicaceae and Limnanthaceae did not have a matrix model in COMPADRE (SI 1). In general, the number of species studied within each family that have a population model currently included in COMPADRE is low (i.e., ranging from 1 species in 15 Families to 11 species in 1 Family).

### Phylogenetic signal in demographic traits

We evaluated whether demographic traits present a phylogenetic signal or evolved independently of the phylogeny. We found that for most traits the phylogenetic signal had intermediate Pagel's *λ* values between about 0.2 and 0.3 (Table 1). Pagel's *λ* was lowest for *L*_*α*_; the median of the posterior distribution (*η*) was 0.17 (0.00:0.38, 95% credible intervals [CI]) and highest for $R_{o}$; *η* = 0.97 (0.71:0.99, 95% credible intervals [CI]).

**Table 1:** Phylogenetic signal in demographic traits of vascular plants in the United States

Trait	*λ*	Lower 95% HPD	Upper 95% HPD	ESS
Ro	0.97	0.71	0.99	56 281
*L*_max_	0.22	0.01	0.44	213 040
*H*	0.23	0.00	0.51	242 091
*L*_mean_	0.29	0.05	0.52	244 834
*L*_*⍺*_	0.17	0.00	0.39	173 487
*S*	0.26	0.00	0.57	294 477

*Note*: Phylogenetic signal was estimated using Pagel's *λ* under a Bayesian framework. Reported *λ* values represent the median values obtained from the posterior distribution.

Abbreviations: ESS, effective sample size; HPD, highest posterior density interval.

### Cluster analysis for herbaceous species

**Figure 2:**
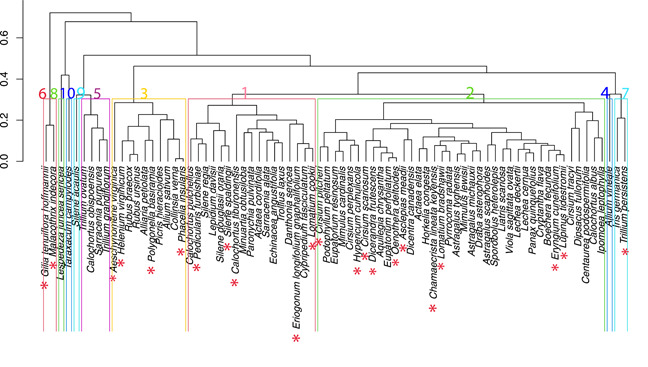
Hierarchical cluster. Hierarchical cluster based on six life‐cycle traits using the Bray–Curtis association distance coefficient and the average linkage method. Traits were normalized with Equation 3. The cutting point for the cluster development was 0.3. Color numbers represent the cluster identity; in total 10 clusters were formed. Red asterisk highlights the listed species. Seventy‐seven plant species in total and 22 listed species are shown

The hierarchical cluster analysis for the 77 species that fulfilled the selection criteria generated 10 clusters that represent 30 families. Among the clusters generated, significant differences were observed between Clusters 1 and 2, 1 and 3, 2 and 3, and 2 and (Figures 2, S2). Of those significantly different pairs, all clusters included listed species except for Cluster 5, which did not include any listed species. Most listed species were included in Cluster 2 (11 species), which was also the cluster with the largest number of species (38 species total and 13 species each from a different family; Figures 2, S3). Cluster 6, though not significantly different from any other cluster (SI 2), included two listed species (*Gilia tenuiflora hoffmannii* and *Malachothrix indecora*; Figure 2).

**Figure 3:**
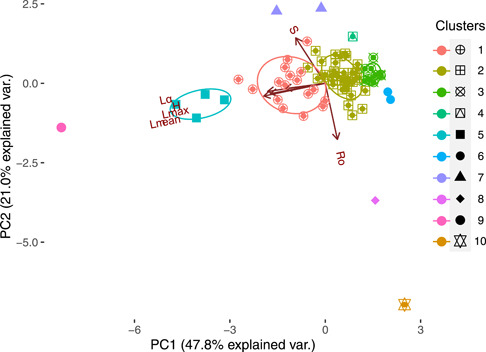
Principal component analysis biplot. Ordination of the species used in the hierarchical cluster (listed and nonlisted). Traits were normalized with Equation (3). Different colors and shapes represent the cluster identity. Net reproductive rate ($R_{o}$), longevity ($L_{\max}$), survivorship curve type (H), mean life expectancy ($L_{\text{mean}}$), age at first reproduction ($L_{\alpha }$), and degree of iteroparity (S)

The PCA showed that the first three principal components explained 81.1% of the total variance in our dataset (PC1 = 47.8%, PC2 = 21%, PC3 = 12.3%; SI 4). The four significantly different clusters, that is, Clusters 1–3, and 5, observed in the cluster analysis, as well as Cluster 6, were aligned over the first component (Figures 3, S2). The loadings of this component were highest for $L_{\alpha }$, $L_{\max}$, and $L_{\text{mean}}$, indicating that the clusters falling along PC1 are ordinated according to the values of $L_{\alpha },$ $L_{\max}$, and $L_{\text{mean}}$ (specifically, Cluster 5 > Cluster 1 > Cluster 2 > Cluster 3) (SI 4). This arrangement of traits describes the continuum of the pace of life, where at one extreme are long‐lived species that reproduce late in life (Clusters 5 and 1), followed by species with intermediate traits (Cluster 2), and at the other extreme short‐lived species that reproduce early in life (Cluster 3). Clusters 1–3 and 5 did not vary across PC2. This component exhibited high loadings for $R_{o}$ and S, which were negatively correlated. In general, the species in these clusters exhibit similar values for $R_{o}$ ($R_{o}$ < 20) and S (S=1.5,0.9,0.3,1.7), whereas, Clusters 7–10, although not significantly different in the PERMANOVA (SI 2), were widely distributed across the second component. S was highest in Cluster 7 > Cluster 9 > Cluster 6 > Cluster 10 > Cluster 8 (S range =  [−2,11.5]) and $R_{o}$ was highest in Cluster 10 > Cluster 8 > Cluster 9 > Cluster 6 > Cluster 7 ($R_{o}\text{range}=[1839])$. The third component was driven by the survivorship in which only Clusters 2 and 3 and some species of Cluster 1 differed.

### Elasticity analysis across clusters

**Figure 4:**
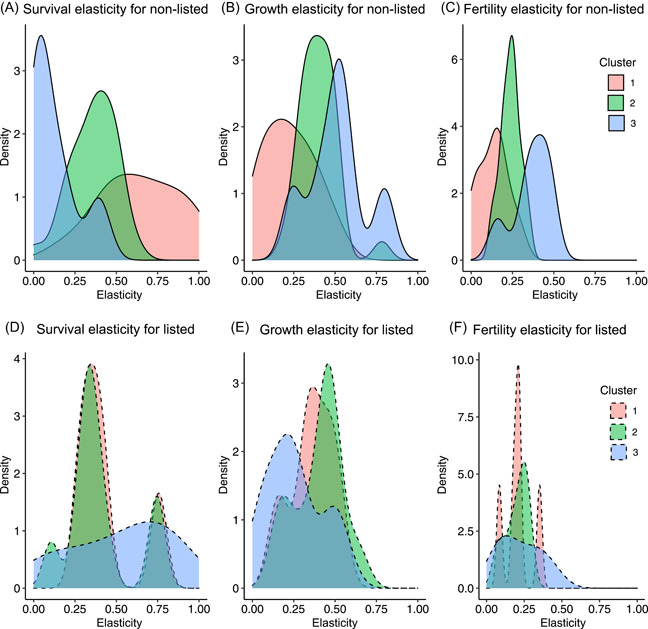
Density plot of elasticities across clusters. Smoothed histograms of each grouped elasticity across Clusters 1–3 for both listed (dashed lines) and nonlisted species (solid lines). The top row illustrates nonlisted species, and the bottom row shows listed species. Cluster 1 is pink, Cluster 2 is green, and Cluster 3 is blue. Elasticity values are on the *x*‐axis. (A) Survival elasticity for nonlisted species. (B) Growth elasticity for nonlisted species. (C) Fertility elasticity for nonlisted species. (D) Survival elasticity for listed species. (E) Growth elasticity for listed species. (F) Fertility elasticity for listed species. Peaks of the plots indicate the value that has the highest frequency in the data set

In general, elasticity values for the three processes analyzed were similar between listed and nonlisted species only in Cluster 2, where species exhibited low variability in each of the elasticity metrics (Figure 4). In this cluster, survival (Figure 4A,D) and growth (Figure 4B,E) elasticity values were ~0.4 (SI 5) and fertility elasticity values (Figure 4C,F) were ~0.2 for plants of different conservation statuses (SI 5). Marked differences in elasticities between listed and nonlisted species were observed in Cluster 3. For example, in Cluster 3 survival elasticity was overall lower in nonlisted species than in listed species (~0.1 vs. ~0.7; Figure 4A,D; the same pattern was observed when the median values were calculated SI 5) with *Aeschynomene virginica* exhibiting the largest survival elasticity (0.8), followed by *Helenium virginicum* (0.6), and *Phacelia insularis* (0.5) (SI 6G,I). Growth and fertility elasticities were higher in nonlisted species than in listed species for Cluster 3 (Figures 4B,C,E,F, S5). Among the listed species, growth elasticity was highest in *Lupinus tidestromii* and fertility elasticity was highest in *Phacelia insularis* and *Polygonella basiramia* (SI,6e, h, i). These marked differences contributed to the high variability in the elasticity values in Cluster 3. In Cluster 1, survival elasticity was higher overall and more variable in nonlisted species than in listed species (~0.6 vs. 0.3), but the general pattern was similar for plants of different conservation statuses where survival and growth elasticity were higher than fertility elasticity (Figures 4A, S5).

Survival elasticities for nonlisted species differed among the three clusters. In Cluster 1, most species exhibited high elasticity values, species in Cluster 2 exhibited intermediate values, and species in Cluster 3 exhibited the lowest values of survival elasticity (Figures 4A, S5). Survival elasticities for listed species across clusters were similar between Clusters 1 and 2 (SI 5; two peaks emerged, at ~0.3 and ~0.75; Figure 4D). Listed species from Cluster 3 spanned a wide range of elasticity values (0.1, 0.81) but most species exhibited elasticity values around 0.75 (Figure 4D).

Growth elasticities for nonlisted species were highest in Cluster 3 (~0.5), followed by Cluster 2 (~0.4) and Cluster 1 (~0.2) (Figures 4B, S5). In listed species, the pattern was different, where Cluster 2 had the highest elasticity (~0.4), followed by Cluster 1 (~0.3) and Cluster 3 (~0.2) (Figures 4E, S5).

Fertility elasticities in nonlisted species were highest in Cluster 3 (~0.4), followed by Cluster 2 (~0.2) and Cluster 1 (~0.1) (Figures 4C, S5). In listed species, fertility elasticities were low in the three clusters (~0.2). Clusters 2 and 1 were similar and slightly higher than elasticities in Cluster 3 (Figures 4C, S5).

## DISCUSSION

Our analysis showed that even with few matrix population models available for listed plant species, comparisons of life‐history traits and elasticities can identify patterns that may help to understand why some species are more susceptible to perturbations (e.g., from herbicide exposure) than others. Such analyses may inform the future selection of representative species for population modeling in the context of pesticide risk assessment. Among our limited sample of species, some clear differences were observed in their age at first reproduction and longevity (Cluster 1 or “long‐lived” > Cluster 2 or “intermediate” > Cluster 3 or “short‐lived”) but not in their reproductive output. We found that most of the traits showed a moderate phylogenetic signal, indicating that close relatives are somewhat more similar in trait values than distant relatives. The exception to this was for $R_{o}$, which had a very high phylogenetic signal, indicating that closely related species are much more similar for this trait than more distantly related species (Molina‐Venegas & Rodriguez, 2017). The reasons for this are not entirely clear, and though beyond the scope of the present study, this result deserves further exploration. The fact that we observed relatively low variability in $R_{o}$ among clusters may have influenced this result. Additionally, we did not include a trait related to seed dispersal as this trait is not included in the COMPADRE database. Since widespread seed dispersal would likely reduce extinction risk, adding this trait to future analyses could improve species vulnerability assessments.

Our elasticity analysis showed that fertility elasticities were generally lower than survival or growth elasticities, which is consistent with other studies (Pfister, 1998; Rueda‐Cediel et al., 2019). Nonlisted and listed species in Cluster 2, which occupy an intermediate position in the continuum of the pace of life, exhibit greater similarity compared to species in Clusters 1 (long‐lived species) and 3 (short‐lived species). For this specific life‐history category, population models of nonlisted species may be representative of listed species and help to inform risk assessments and prioritize the management of listed species. A good starting point to identify representatives for listed species lacking detailed demographic data could be to characterize ranges for the traits that defined Cluster 2, for example, longevity and age at first reproduction.

The substantial variability in the elasticity values observed in Clusters 1 and 3 make it more challenging to identify suitably representative species on this basis. One approach could be to select species from the higher end of the elasticity distributions. For example, among the long‐lived species (Cluster 1), *Minuartia obtusiloba* and *Paronychia pulvinata* exhibit the highest values of survival elasticity. These species may provide a sensitive representative for the species in this cluster when trying to evaluate the potential effects of herbicides that target plant survival.

The clusters are based on the demographic traits we calculated. As explained above, the plant species in Clusters 1–3 differed in their longevity. For nonlisted species (Figure 4A–C), differences among clusters in survival elasticities are consistent with the literature with elasticity positively correlated with lifespan. Likewise, fertility elasticities were greater in short‐lived than long‐lived species, but in all clusters lower than survival elasticities. Again, this is consistent with the literature. However, the listed species show very different patterns (Figure 4D–F). Whether this is due to the small sample size or some other feature of the listed species is not clear at this time

### Demographic data gaps across US plants: A phylogenetic perspective

The homogeneous distribution of listed species across the trimmed phylogeny reflects the multiple threats that affect plant biodiversity (e.g., habitat fragmentation, climate change, and fire), which are not specific to any taxonomic grouping. The less homogeneous distribution of plant species with population models currently in COMPADRE may reflect limited knowledge of plant life history or specific research interest in plant demography of certain families or species. Identification of these data gaps may help guide researchers, managers, and decision‐makers to select representative species or families to study.

In terms of families, our data sample represents ~38% of the plant families for angiosperms and gymnosperms worldwide, and out of that, our sample of listed species represents ~15% of families of plant species worldwide. Although this is a relatively small percentage globally, the United States has a proportionally high percentage of species of conservation concern (Liu et al., 2019).

### Cluster analysis for herbaceous species

The even distribution of listed species in the hierarchical cluster suggests that there are no particular combinations of life‐history traits that make it more likely that a species will be listed. The similarity in life‐history traits between listed and nonlisted species from different listing protocols has been demonstrated (Rueda‐Cediel et al., 2019) and is perhaps not surprising given the ambiguity of the listing criteria in the ESA. Listing categories are typically not determined by relevant life‐history traits; rather they are based on the number of observed populations and visible declines (Boyd et al., 2016; Evans et al., 2016; Thompson et al., 2018). In our analysis, species belonging to significantly different clusters—also most of the listed species in the analysis—differed primarily in average longevity, maximum longevity, and age at first reproduction. These traits determine the fast–slow continuum, which is one of the two main axes describing plant life histories (Salguero‐Gómez et al., 2016). The second axis identified in our analysis was the reproductive strategy axis (Salguero‐Gómez et al., 2016), but the significantly different clusters did not vary along this axis. In other words, there was zero to low variability in net reproductive output or frequency of reproductive events across the listed and nonlisted species in these Clusters (1–3). This lack of variability in the second axis across species may be due to various factors. For instance, the degree of iteroparity was fairly similar across all the species analyzed, and the majority of species, belonging to a single cluster (i.e., Cluster 2), were characterized by a low net reproductive rate. However, the fact that some species can be differentiated from others by their life‐history traits raises the possibility to develop trait‐based models to study the potential effects of chemicals and other stressors on plant population dynamics as has been done for graminoids (Quétier et al., 2007), tropical trees (Visser et al., 2016), and aquatic macroinvertebrates (Rico & Van Den Brink, 2015; Van den Berg et al., 2019), where groups of species sharing particular sets of traits also share responses to specific stressors, including pesticides.

### Elasticity analysis across clusters

The elasticity analysis showed that species in Cluster 2 respond similarly to perturbations of their survival, growth, and fertility. This indicates that the population growth rate is affected in a similar way by small changes in any of the three processes. Species in this cluster are characterized by intermediate trait values of longevity and age at first reproduction, indicating that they do not fall in any of the extremes of the pace of the life continuum. Species like *Actaea cordifolia, Minuartia obtusiloba, Paronychia pulvinate*, and *Calochortus tiburonensis*, among others, that reproduce late in life and have a long‐life span, tend to favor growth at the expense of reproductive output (Vico et al., 2016). At the other extreme of the continuum, species like *Collinsia verna, Aeschynomene virginica*, and *Phacelia insularis*, among others, that have short life spans and reproduce early, allocate more energy toward reproduction (Wenk & Falster, 2015). Species in these two extremes would be expected to exhibit a marked difference between the elasticity of survival and growth and the elasticity of fertility as can be seen in Clusters 1 and 3.

The differences observed in Clusters 1 and 3 between species of different conservation statuses may be due to variability in the reproductive potential of the species and age at first reproduction. For example, most of the listed species in Cluster 1 take longer (>4 years) to reach reproductive age than the nonlisted species (<4 years), resulting in higher growth elasticity. Therefore, these species would be more vulnerable to changes in growth and reproduction compared to nonlisted species that reproduce earlier and have a slightly higher net reproductive rate. However, it is important to remember that there may be intraspecific variability in traits among different populations of the same species (e.g., due to environmental and habitat influences). Whereas using differences in life‐history traits among species or species groups to assess relative vulnerability are likely to be broadly robust, a high level of precision should not be assumed.

Characterizing the life‐history variation of listed species, with the aid of phylogeny and comparative analyses that bring together listed and nonlisted species, provides a useful tool to prioritize species in terms of their likely vulnerability to demographic perturbations thus informing the risk assessment and management process for a wider range of species. Our analysis showed that US plant life histories can be arranged across the continuum of the pace of life, and that listed and nonlisted species with intermediate life histories tend to share vulnerabilities to perturbations to core life‐history processes. This suggests that we should be able to identify representative species from this group to be used in population models applied in a risk assessment context. For species at either end of the pace of life continuum, in which there seem to be some differences in their sensitivity to perturbations between listed and nonlisted species, selection of representatives is more challenging. In such cases, a conservative approach would be to select high‐elasticity species as the more vulnerable representatives for further model development. Alternatively, species at either end of the elasticity range could be modeled to capture the inherent uncertainties associated with the elasticity variability.

Our analysis used matrix population models to categorize listed and nonlisted species according to their life‐history traits and inform the selection of representative species for population modeling in the context of pesticide risk assessments. Even though matrix population models for these species already exist, these do not incorporate the relevant processes and detail required for application to pesticide risk assessment (Galic et al., 2010; Schmolke et al., 2010). Population models for species identified as representative for specific clusters could further be developed that contain the relevant information and are tailored for pesticide risk assessments. The choice of model structure and the inclusion of relevant processes and features will depend on the questions the ERA is designed to address (Accolla et al., 2021, Raimondo et al., 2018).

## CONCLUSION

Population models represent powerful tools for listed species assessments because they can integrate pesticide exposure as well as species sensitivity data and life‐history traits to produce quantitative projections of population trends. Developing population models for all listed species of terrestrial plants would not only be impractical due to the sheer number of species listed as threatened or endangered but impossible due to the scarcity of relevant data for the majority of listed plant species. Basing the selection of representative species in lieu of data on systematic life‐history analyses helps to ensure that population models representing listed species are both representative and protective.

## CONFLICT OF INTEREST

The authors declare no conflicts of interest.

## DISCLAIMER

The peer review for this article was managed by the Editorial Board without the involvement of V. Forbes.

## Supporting information

This article includes online‐only Supporting Information.

SI 1. Families represented in the sample of listed species and species present in COMPADRE. Red indicates the presence in the list of species listed under the ESA. Green indicates the presence in the COMPADRE database.

**SI 2**. Permutational multivariate analysis of variance (PERMANOVA). Permutational multivariate analysis of variance (PERMANOVA) results are based on Bray–Curtis dissimilarities of data normalized with equation 3.

**SI 3**. Families represented in the hierarchical cluster. The red color indicates the species' presence in the list of species listed under the ESA. The green color indicates the species' presence in the COMPADRE database.

**SI 4**. Principal component analysis results. Net reproductive rate (R__o_), longevity (L__max_), survivorship curve type (H), mean life expectancy (L__mean_), age at first reproduction (L__α_), and degree of iteroparity (S).

**SI 5**. Table with median elasticity values and ranges across Clusters 1, 2, and 3. Median elasticity values for each of the three processes were evaluated across clusters, which were significantly different in the PERMANOVA and across conservation listings. Ranges are reported in square brackets. The numbers of species for each cluster and listed category are reported in parentheses. No, refers to species that are not listed, and yes refers to species that are listed in the ESA.

**SI 6**. Scatter plot of elasticity values. Each row panel represents a cluster. The top panel is Cluster 1, the middle panel is Cluster 2, and the bottom panel is Cluster 3. Listed species (pink) nonlisted species (green).

## Data Availability

Data are accessible from the COMPADRE Plant Matrix Database at https://www.compadre-db.org/ and from The PLANTS Database at http://plants.usda.gov.
