# Supplementary figures and images for: Using life‐history trait variation to inform ecological risk assessments for threatened and endangered plant species
Source: Integr Environ Assess Manag. 2022 May 24;19(1):213–23. doi: 10.1002/ieam.4615 (PMC10083932; doi:10.1002/ieam.4615)

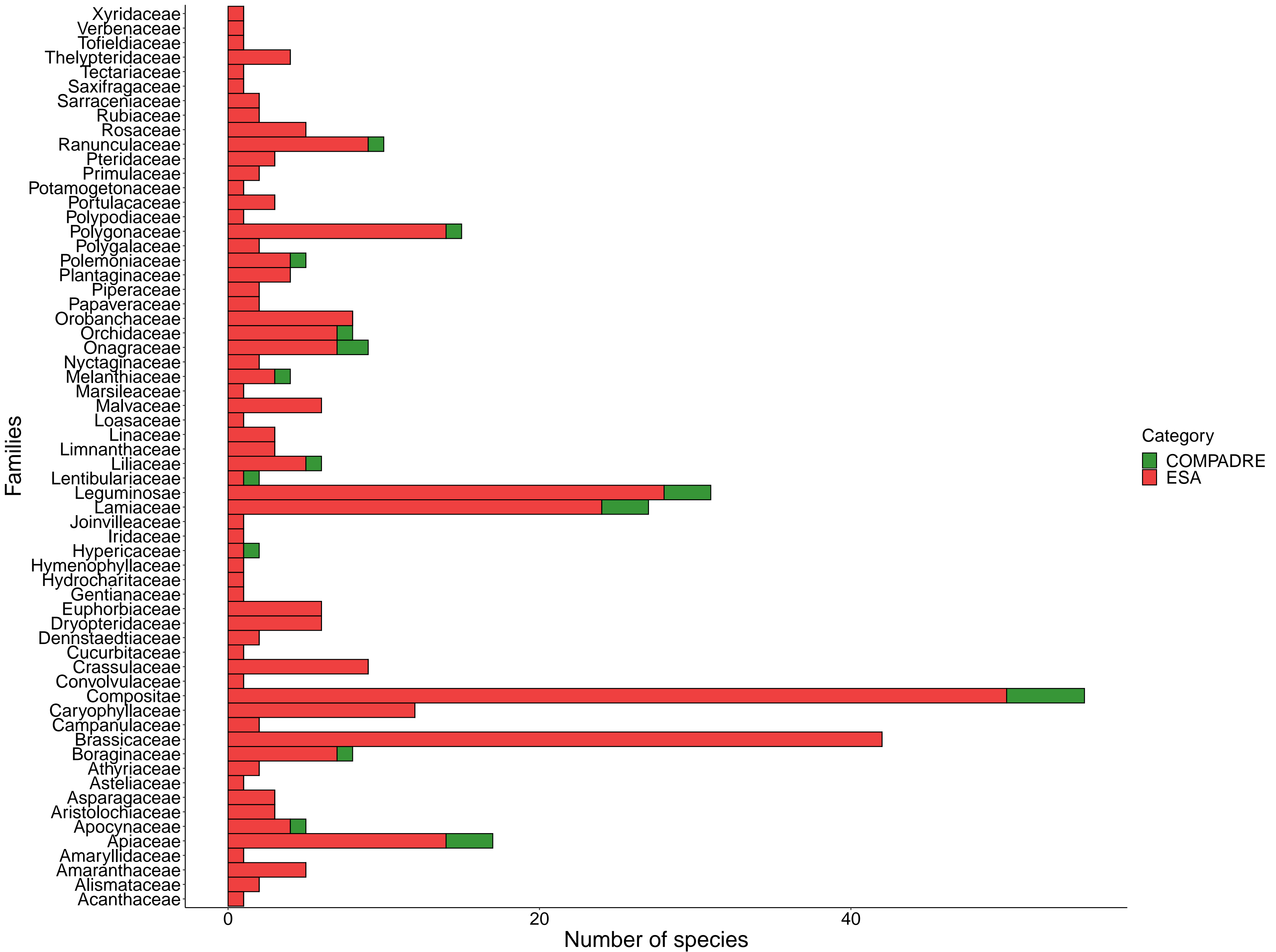

Supplement: Supplementary file 1 — SI 1. Families represented in the sample of listed species and species present in COMPADRE. Red indicates the presence in the list of species listed under the ESA. Green indicates the presence in the COMPADRE database. [file IEAM-19-213-s002.pdf]

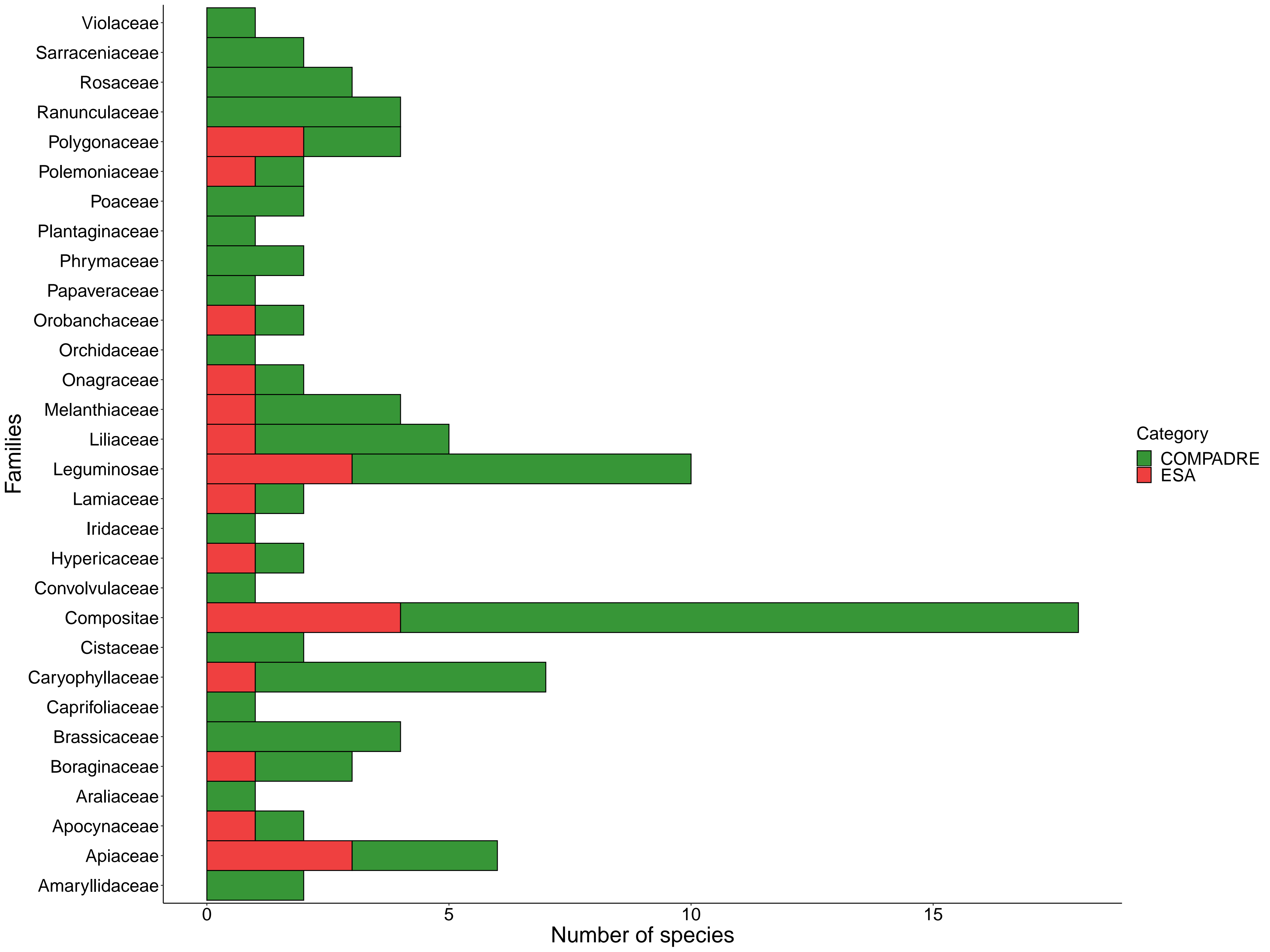

Supplement: Supplementary file 3 — SI 3. Families represented in the hierarchical cluster. The red color indicates the species' presence in the list of species listed under the ESA. The green color indicates the species' presence in the COMPADRE database. [file IEAM-19-213-s001.pdf]

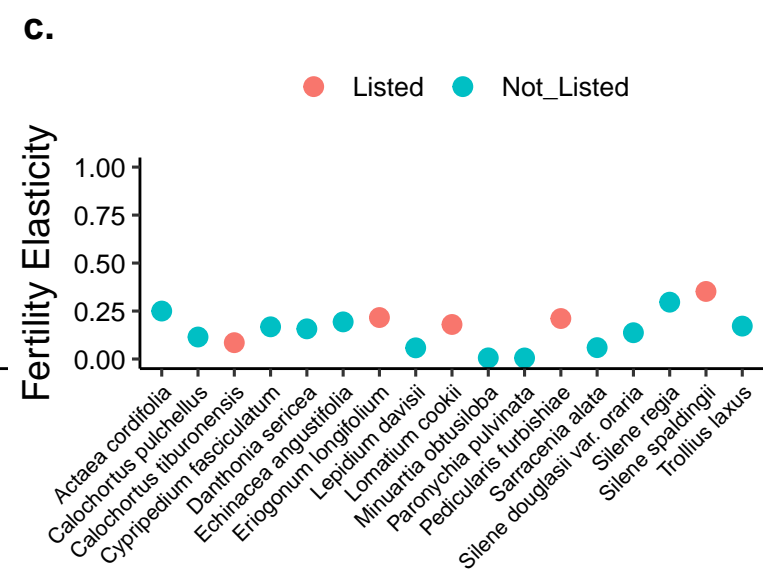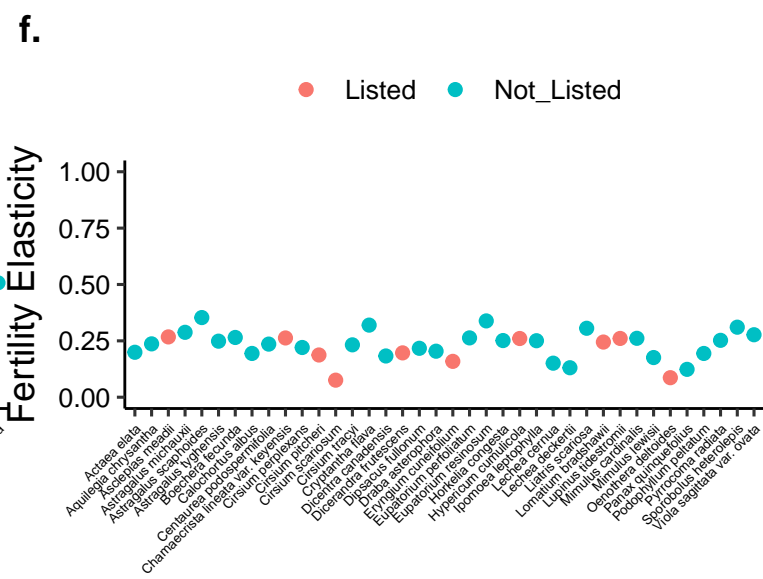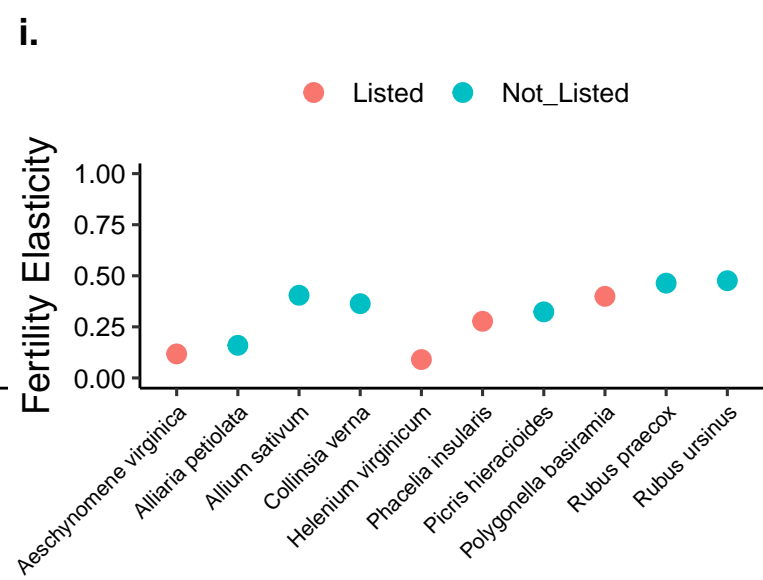

Supplement: Supplementary file 6 — SI 6. Scatter plot of elasticity values. Each row panel represents a cluster. The top panel is Cluster 1, the middle panel is Cluster 2, and the bottom panel is Cluster 3. Listed species (pink) nonlisted species (green). [file IEAM-19-213-s006.pdf]
