# Supplementary material for: Using life‐history trait variation to inform ecological risk assessments for threatened and endangered plant species
Source: Integr Environ Assess Manag. 2022 May 24;19(1):213–23. doi: 10.1002/ieam.4615 (PMC10083932; doi:10.1002/ieam.4615)
Supplement: Supplementary file 2 — SI 2. Permutational multivariate analysis of variance (PERMANOVA). Permutational multivariate analysis of variance (PERMANOVA) results are based on Bray–Curtis dissimilarities of data normalized with equation 3. [file IEAM-19-213-s005.pdf]

pairs	F.Model	R2	p.value	p.adjusted
1 vs 2	246.4416	0.823004	0.001	0.039
1 vs 3	244.7662	0.907327	0.001	0.039
2 vs 3	251.8204	0.845544	0.001	0.039
2 vs 5	334.9279	0.893313	0.001	0.039
1 vs 5	94.07541	0.831971	0.002	0.078
3 vs 5	464.0072	0.97479	0.002	0.078
2 vs 6	248.1272	0.867192	0.003	0.117
2 vs 7	246.4974	0.866431	0.003	0.117
1 vs 7	76.67889	0.818529	0.007	0.273
1 vs 6	95.59116	0.849011	0.008	0.312
3 vs 7	644.7116	0.984726	0.015	0.585
3 vs 6	490.1837	0.980007	0.02	0.78
2 vs 8	155.8635	0.808154	0.023	0.897
2 vs 10	203.169	0.845942	0.025	0.975
1 vs 4	28.08389	0.637056	0.047	1
1 vs 8	46.86786	0.745498	0.048	1
1 vs 9	43.1011	0.729277	0.056	1
1 vs 10	57.20195	0.781427	0.059	1
2 vs 4	52.91439	0.588497	0.026	1
2 vs 9	182.7267	0.831609	0.03	1
3 vs 4	60.72602	0.870923	0.098	1
3 vs 8	521.0543	0.983021	0.09	1
3 vs 9	688.7091	0.987101	0.112	1
3 vs 10	676.2472	0.986866	0.085	1
4 vs 5	24.44675	0.890697	0.2	1
4 vs 6	3477.337	0.999713	0.333333	1
4 vs 7	70.88823	0.98609	0.333333	1
5 vs 6	51.4355	0.927844	0.066667	1
5 vs 7	47.00123	0.921571	0.066667	1
5 vs 8	40.54304	0.931103	0.2	1
5 vs 9	32.88143	0.916391	0.2	1
5 vs 10	71.22157	0.95958	0.2	1
6 vs 7	71.03971	0.972618	0.333333	1
6 vs 8	2473.561	0.999596	0.333333	1
6 vs 9	7352.465	0.999864	0.333333	1
6 vs 10	5527.752	0.999819	0.333333	1
7 vs 8	24.99387	0.961529	0.333333	1
7 vs 9	50.80493	0.980697	0.333333	1
7 vs 10	64.95133	0.984837	0.333333	1
4 vs 8	NA	1	NA	NA
4 vs 9	NA	1	NA	NA
4 vs 10	NA	1	NA	NA
8 vs 9	NA	1	NA	NA
8 vs 10	NA	1	NA	NA
9 vs 10	NA	1	NA	NA
