# Supplementary material for: Using life‐history trait variation to inform ecological risk assessments for threatened and endangered plant species
Source: Integr Environ Assess Manag. 2022 May 24;19(1):213–23. doi: 10.1002/ieam.4615 (PMC10083932; doi:10.1002/ieam.4615)
Supplement: Supplementary file 4 — SI 4. Principal component analysis results. Net reproductive rate (R_o), longevity (L_max), survivorship curve type (H), mean life expectancy (L_mean), age at first reproduction (L_α), and degree of iteroparity (S). [file IEAM-19-213-s003.pdf]

	PC1	PC2	PC3	PC4	PC5	PC6
R_o	0.108	-0.751	0.078	-0.644	0.047	-0.020
L_{max}	-0.486	-0.138	-0.194	-0.010	-0.802	0.251
H	-0.353	-0.070	0.917	0.134	0.034	0.101
L_{mean}	-0.545	-0.170	-0.160	0.123	0.153	-0.781
L_{α}	0.511	-0.108	-0.293	0.030	0.574	0.558
S	-0.262	0.609	0.061	-0.742	0.025	-0.075
**Standard deviation**	1.694	1.122	0.861	0.778	0.637	0.348
**Proportion of variance**	0.478	0.210	0.124	0.101	0.068	0.020
**Cumulative proportion**	0.478	0.688	0.811	0.912	0.980	1.000
