# Supplementary material for: Using life‐history trait variation to inform ecological risk assessments for threatened and endangered plant species
Source: Integr Environ Assess Manag. 2022 May 24;19(1):213–23. doi: 10.1002/ieam.4615 (PMC10083932; doi:10.1002/ieam.4615)
Supplement: Supplementary file 5 — SI 5. Table with median elasticity values and ranges across Clusters 1, 2, and 3. Median elasticity values for each of the three processes were evaluated across clusters, which were significantly different in the PERMANOVA and across conservation listings. Ranges are reported in square brackets. The numbers of species for each cluster and listed category are reported in parentheses. No, refers to species that are not listed, and yes refers to species that are listed in the ESA. [file IEAM-19-213-s004.pdf]

Cluster	ESA listed	Survival	Growth	Fertility
1	No	0.6495	0.2092	0.1471
	(12)	[0.1975,0.9650]	[0.0291,0.5062]	[0.0055,0.2962]
	Yes	0.3742	0.3592	0.2110
	(5)	[0.2750,0.7569]	[0.1579,0.5139]	[0.0852,0.3521]
2	No	0.3768	0.4006	0.2426
	(28)	[0,000,0.6118]	[0.1941,0.7830]	[0.1236,0.3533]
	Yes	0.3531	0.4527	0.2207
	(10)	[0.1062,0.7494]	[0.1709,0.6331]	[0.0757,0.2675]
3	No	0.0706	0.5143	0.3840
	(6)	[0.0157,0.3938]	[0.2427,0.7985]	[0.1597,0.4760]
	Yes	0.5804	0.2360	0.1974
	(4)	[0.1004,0.8198]	[0.0626,0.5003]	[0.0900,0.3993]
